# Exploring soybean metabolic pathways based on probabilistic graphical model and knowledge-based methods

**DOI:** 10.1186/s13637-015-0026-5

**Published:** 2015-06-20

**Authors:** Jie Hou, Gary Stacey, Jianlin Cheng

**Affiliations:** 1 Department of Computer Science, University of Missouri, Columbia, MO 65211 USA; 2 Divisions of Biochemistry and Plant Science, National Center for Soybean Biotechnology, C. Bond Life Science Center, University of Missouri, Columbia, MO 65211 USA

**Keywords:** Soybean, Gene expression data, RNA-seq, Metabolic pathway, Bayesian network, KEGG database

## Abstract

Soybean (*Glycine max*) is a major source of vegetable oil and protein for both animal and human consumption. The completion of soybean genome sequence led to a number of transcriptomic studies (RNA-seq), which provide a resource for gene discovery and functional analysis. Several data-driven (e.g., based on gene expression data) and knowledge-based (e.g., predictions of molecular interactions) methods have been proposed and implemented. In order to better understand gene relationships and protein interactions, we applied probabilistic graphical methods, based on Bayesian network and knowledgebase constraints using gene expression data to reconstruct soybean metabolic pathways. The results show that this method can predict new relationships between genes, improving on traditional reference pathway maps.

## Introduction

Soybean (*Glycine max* L. Merr.) is recognized as an important food source for humans and animals because of its relatively high protein and oil ingredients. As one major species of the legume family, soybean contains high-quality protein which is a fundamental requirement, providing complete protein that contains all the essential amino acids that people need. Soybean is also considered “heart healthy” since soybean protein intake can significantly decrease serum (blood) cholesterol and low-density lipoprotein (LDL) levels, contributing to a reduced risk of coronary heart disease [1, 2].

A remarkable achievement in soybean research was the completion of the genome sequence (http://www.phytozome.net/soybean), which provided the basis for a variety of detailed, genome-wide studies, including completion of a transcriptome atlas based on RNA-seq analysis of different tissues [3, 4]. The availability of this transcriptome data facilitates more detailed studies of soybean gene function (e.g., [5]).

In order to visualize and analyze large-scale experimental gene expression data, especially to elucidate gene–gene and protein–protein interactions, gene and protein expression data are commonly mapped to reference metabolic pathways, which provides a context for understanding the functional response of the plant to a given treatment. Metabolic pathways are designed to represent the chemical reactions among a set of small molecules in a cell within one organism. Therefore, reconstruction of metabolic pathways from protein and gene expression data can help researchers discover new, fundamental biological functions for a particular network. Although more and more plant genome sequences are becoming available, there is still need for improved methods for metabolic pathway reconstruction to support functional studies.

In order to reconstruct a traditional metabolic pathway for a given species (e.g., those provided by the KEGG database [6, 7]), the annotated genes and their encoded protein products are integrated with the reference metabolic pathways in the KEGG database. The gene product sequences are mapped to the reference pathway using the KEGG Automatic Annotation Server (KAAS) [8] based on sequence homology to similarly mapped sequences from well-annotated reference genomes. Each gene is assigned one KEGG orthology number (KO number) with the highest ranking based on the functional annotation in KAAS and scoring orthology groups by probability and heuristics. The association between KO numbers leads to placement of the gene products into curated pathways. In order to improve on these methods, providing more potential and valuable interactions between genes and proteins, we applied the Bayesian network to construct probabilistic graphical networks. By way of example, we used knowledgebase constraints to improve the prediction efficiency and accuracy to reconstruct metabolic pathways for soybean [9].

## Method

### Pathway construction workflow

**Fig. 1:**
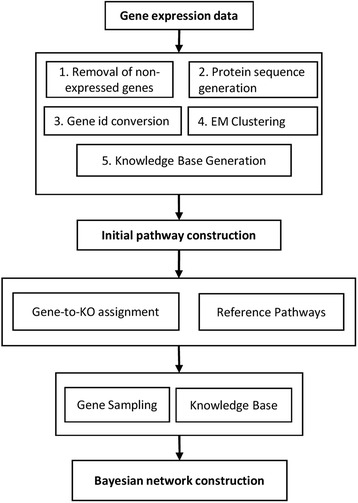
The workflow of Bayesian network pathway construction. The procedure started with preprocessing of the gene expression data, including removal of non-expressed genes, protein sequence generation, gene ID conversion, gene data clustering, and knowledgebase generation. The protein sequence for all genes was then mapped to the KEGG database for initial pathway construction. The Bayesian network method was used to predict new metabolic pathways by integration of the gene-to-KO assignments, reference pathways, gene sampling, and knowledge constraints

The metabolic pathways were constructed by integrating the available soybean gene expression data, KEGG reference pathways, and the probabilistic network modeling method with knowledgebase constraints. The data- and knowledge-driven methods are shown in Fig. 1. The first step is to preprocess the gene expression data, such as removing genes showing no apparent expression, protein sequence prediction, gene data clustering, and knowledgebase generation. After a translated protein sequence is generated for each gene, these proteins can be mapped to the KEGG reference pathways using the KAAS mapping tool for initial pathway construction. This mapping information is then integrated with newly sampled genes from gene clustering, and knowledge constraints for KO relations in KEGG. The gene expression data is fed into the Bayesian network model to predict gene–gene interaction networks. Finally, related chemical compounds are added to the network to better represent the metabolic pathway. The detailed introduction for each step is shown in the following sections.

### Data preprocessing

#### Removal of non-expressed genes

The published RNA-seq gene expression data, representing 14 soybean tissue-specific conditions, were normalized to counts per million reads (CPM) [3]. The CPM normalization was implemented using the Bioconductor package edgeR [10] within the R statistical programming language. The genes with a retrieved CPM value above one in at least one condition were kept for further analysis, while those genes showing no apparent expression in any of the 14 conditions were removed from the dataset.

#### Protein sequence generation

Accurate gene translation is essential for developing the initial reference pathway maps. Protein sequences available for the annotated soybean genome were extracted from the Soybean Knowledge Base (SoyKB) [11, 12]. The KEGG pathway database utilizes Entrez IDs for each soybean gene and, therefore, the SoyKB Glyma-format ID was converted to EntrezGene ID (ex., GLYMA01G00300.1 ↔ 100781438) using BioMart [13]. The metabolic pathways were built by combining all of the knowledge above (i.e., protein annotation and associated gene expression values).

#### Gene clustering

Metabolic pathways are graphical representations of cellular processes in the KEGG database. Each reference pathway is composed of a network of enzymes and a set of genes that are functionally related in terms of predicted cellular and molecular functions based on experimental knowledge [6, 7]. One assumption of our metabolic pathway reconstruction method is that genes that share similar gene expression patterns over a set of experiments are more likely to be involved in the same reference metabolic pathway. Therefore, we used the Expectation-Maximization-based clustering algorithm on the data sets [14]. This clustering algorithm is Gaussian mixture model based and the clustering number is determined by cross validation to improve the consistency and robustness for gene selection.

#### Relation knowledgebase construction

Information on all the existing relationships among KO identifiers (ortholog group) for all the mapped genes in all pathways can be collected from the KEGG database [6, 7]. Such a relational knowledgebase includes the relation and reaction among orthologous groups, such as the directional relations, the relationship type within protein–protein interactions and protein–compound interactions, as well as related chemical reactions.

In order to generate the relation knowledgebase, based on the gene-to-KO assignments and the list of pathway maps with KGML format generated by the method described above in the “Initial pathway construction” section, we extracted all the sets of relations and reactions for mapped KO numbers from the full set of KEGG reference pathways [9]. The relationship between genes can also be predicted based on the relationship between KO numbers.

In order to improve the space searching efficiency and quality for Bayesian network construction, two knowledgebase sets were generated [9]. A gene whitelist was created for Bayesian network prediction consisting of all the relationships between genes. A blacklist was also constructed consisting of all possible gene relations not supported by the knowledgebase and, therefore, excluded from the Bayesian network construction.

### Initial pathway construction

The KEGG database [6, 7] provides 90 graphical diagrams for soybean reference metabolic pathways, which were computationally generated from manually curated pathways based on experimental knowledge of metabolism. Each pathway represents the network structure of chemical compounds, enzyme molecules and enzymatic reactions, where each enzyme is assigned one Enzyme Commission (EC) number to specify enzyme-catalyzed reactions. Each EC number is associated with a KEGG Orthology (KO) number in the KEGG database. The KO number is a unique identifier for matching the genomic information in the GENES database and the gene products (enzyme–enzyme interaction) information in the PATHWAY database. In each reference pathway, rectangle nodes are assigned with the KO identifiers to denote specific enzymes. Once the KO identifiers are assigned to genes in a specific genome, the related organized-specific pathways are generated automatically. A web-based server called KEGG Automatic Annotation Server (KAAS) [8] can automatically assign KO identifiers to genes based on the protein sequence similarities, which enables the reconstruction of initial organism-specific pathways and BRITE hierarchies.

However, since the complete metabolic pathway is separated into a list of subpathways in terms of different cellular, molecular functions, genes mapped to a specific pathway can only represent a small part of relationships in the whole pathway. The traditional mapping method such as KAAS can only predict a small subset of relationships that exist in each reference pathway. In order to address this weakness, we applied the Bayesian probabilistic network method [9] to expand the initially mapped pathways by adding more genes and relationships, taking into account all predicted KO relationships in KEGG. Based on the gene-to-KO assignment and pathway network structure information, the initial pathway can be constructed by matching the genes to the KO identifiers in each pathway.

### Bayesian network pathway construction

After the initial pathways for soybean genes were constructed and the associated knowledgebase built, we expanded the pathways by adding more genes with similar gene expression patterns and new relationships and reactions. The new genes were derived from predicted gene clusters and metabolic pathways predicted through the Bayesian network method taking advantage of all existing relationships between KO numbers in the knowledgebase. The score-based heuristic approach was applied to learn all the possible local structures to enlarge the whole metabolic pathway step-by-step [15].

#### Gene sampling

In order to sample genes that are more likely to be involved in the same pathway, the gene cluster containing more genes than in the initial pathway was assigned the highest probability for sampling. The sampling probability for the remaining clusters was assigned automatically based on the Euclidean distance between each cluster and pivot cluster [9]. The probability assignment follows the criteria that a shorter distance has higher weight for sampling, which means the gene expression values are more similar between genes in two clusters.

#### Bayesian network construction

The Bayesian network approach can be applied to discover casual relationships from gene expression data, which proposes a probabilistic model with joint probability distribution to represent the gene expression patterns for the target genes across the different experimental conditions. Based on the predicted network structure, valuable biological information can be extracted to understand the regulation process among genes. Bayesian network is represented as a directed acyclic graph (DAG), with the gene/protein as nodes and the reactions/relation between genes as directed edges in graphical representation of metabolic pathways. The score function, which is Bayesian Information Criterion (BIC) based, is used to predict networks from gene expression data [15]. The score can be evaluated by adding, removing, and reversing a single edge at each local structure updating step during the network learning process. The greedy hill-climbing algorithm can help find the optimal structure network with a local maximum. After determining the local optimal structure, the new local network is considered as a new node to be used to repeat the sampling procedure to produce a larger pathway network.

Since network learning in a large searching space is time-consuming, we used the knowledge constraints to restrict the network search, resulting in a smaller searching space instead of the overall search space. The concepts of a whitelist and blacklist in Bayesian network were applied. The edges existing in the initial pathways were always present in the graph, serving as a whitelist in the Bayesian network. Based on the sampled genes and existing relationships of orthologous groups (KO numbers) in KEGG, the gene relations that do not exist in the knowledgebase will never be present in the graph, which is served as the blacklist for the Bayesian network.

#### Parsing and editing pathway information

There are several problems that need closer attention during the pathway processing for network construction.

##### Cycle detection and processing in pathway

Metabolic pathway reflects a series of reactions between enzymes, which is often feed-forward reactions with one direction. However, reversible reaction will also exist in the pathway that leads to feedback loops among sets of enzymes. Before feeding the sampled genes from gene cluster combined with initial mapping pathway into Bayesian network reconstruction, the presence of cycles should be conquered in advance since Bayesian network could not handle loops or cycles in graph. During the whitelist generation step for Bayesian network, the gene–gene reactions with direction from the initial pathway that exists in the KEGG knowledgebase were added into whitelist sets iteratively with checking to see if the cycle exists in the current network at each time. If the gene pairs with directed reaction would cause a loop among the current gene network in the whitelist, the edge was not be added. This step generates the initial gene network as a whitelist with no cycles occurring for Bayesian network prediction. In order to incorporate the reaction information from all species in the KEGG database, if the reaction for a gene pair belongs only to soybean, this edge was also excluded from the whitelist set in order to make the initial mapping network more independent. This also helped to validate the performance of our network prediction. After the new network is predicted, the initial mapping network with cycles was amended to the predicted pathway to complete the existing feedback reaction activity.

##### Multi-molecule nodes in a pathway

In the metabolic pathway, multiple proteins may catalyze the same reactions and inhibit or activate the same substrate. Such a set of molecules was grouped together and labeled as one node in the pathway, sharing the same node identifier. In the KGML file for the KEGG pathway, each node is composed of multiple different KO numbers. During the extraction of gene relationships from the initial mapping pathways, the relationships between two nodes in the pathway were assigned to each KO number pair from two nodes to generate the gene–gene relationship. After the gene network was generated by Bayesian network construction, genes belonging to same KO number were grouped together to simplify the network representation.

### Functional enrichment analysis

Protein function prediction software MULTICOM-PDCN [16, 17] was used for function prediction of gene sets from the Bayesian graphical network. A set of Gene Ontology (GO) [18] terms associated with three functional categories (i.e., biological process, molecular function, and cellular components) was predicted for each gene. A Fisher exact test was conducted on each predicted pathway to identify over-represented GO terms, which are significant GO terms associated with the group of genes in the pathway. The significant GO terms identified in each Bayesian network served as a reference to validate the predicted edges among gene sets.

## Results and discussion

### Data description

Our initial input data were RNA-seq gene expression data, representing 14 soybean tissue-specific conditions, including 9 different soybean tissues (root hair cells isolated 84 and 120 h after sowing (HAS), root tip, root, mature nodules, leaves, shoot apical meristem (SAM), flower and green pods [3], as well as 5 additional tissues taken from Libault et al. [19]. This large scale of transcriptomic analysis provided a comprehensive compendium of soybean gene expression. We applied our pathway reconstruction pipeline to this full set of transcriptome data, which contains expression measurements on 69,077 putative annotated soybean genes and 7314 unannotated genes in which 53,175 putative annotated genes were expressed in at least one condition while 15,902 putative annotated genes showed no apparent expression. Genes that were not expressed at all and unannotated were removed from further analysis. Each gene identifier was labeled using the Glyma-format following the convention adopted by the Arabidopsis community [20].

Since one gene can have multiple transcripts or protein sequences due to alternative splicing, we extracted all the transcript variant IDs for the 53,175 expressed genes. For example, gene Glyma20g01000 had only one transcript variant Glyma20g01000.1, while Glyma01g00300 had two transcript variants, Glyma01g00300.1 and Glyma01g00300.2. Protein sequence data were extracted from the Soybean Knowledge Base (SoyKB) [11, 12] providing 35,505 protein sequences; from the expressed 53,175 gene transcripts, protein sequence information for the remaining 17,670 genes was not provided in SoyKB. When dealing with the gene ID conversion process through BioMart [13], BLAST [21, 22] was used to align the transcript sequence against the EntrezGene database and the EntrezGene identifier with high degree of sequence similarity was assigned to transcript ID. Because of this similarity-based mapping method, the same transcript variant might have several different EntrezGene IDs, while several transcript variants might share identical EntrezGene IDs. In such situations, we downloaded the protein sequence information for all soybean genes in the KEGG pathway database and the one-to-one mapping between Glyma-format ID, and EntrezGene ID for each transcript was chosen based on sequence identity. This step removed those transcripts from same gene that their sequences did not match the gene sequences in KEGG database. Finally, the protein sequence information and gene expression value for a total of 26,873 protein-coding genes were built and used for metabolic pathway reconstruction.

**Fig. 2:**
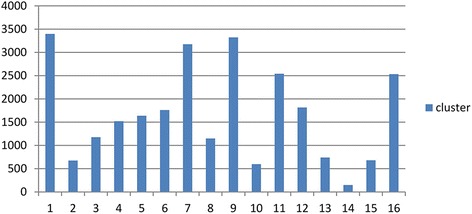
The gene size distribution for 16 clusters. The genes were organized into 16 groups using the Expectation-Maximization clustering method. Genes in the same cluster share a highly similar gene expression pattern, while genes from different clusters have low similarity to each other. These 16 gene clusters were used for gene sampling to enlarge the initial, mapped pathways. *X*-*axis* denotes the index of clusters; *Y*-*axis* denotes the number of genes in each cluster

These 26,873 genes were clustered into 16 clusters using the Expectation-Maximization-based clustering algorithm with an average of 1679 in each cluster. These clusters were then used to enlarge the initial, mapped pathways. The gene size distribution for 16 clusters is shown in Fig. 2.

**Fig. 3:**
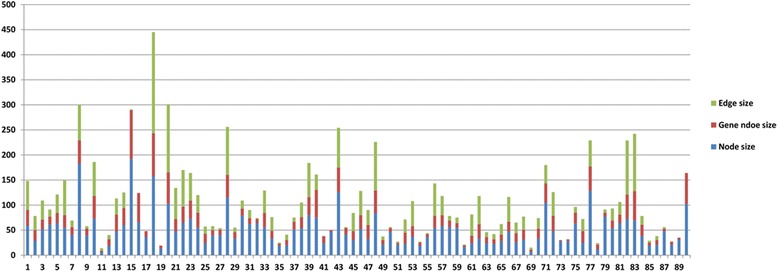
The statistics of nodes and the edges for 90 soybean target pathways. The statistics of nodes and the edge relationships (e.g., ECrel, PPrel, GErel) for 90 soybean target pathways, along with the number of true soybean genes mapped to each target pathway. Among 90 target pathways, there were 9 pathways with no edge relationships between KO-group nodes. Therefore, these were removed from further analysis, leaving 81 pathways for prediction validation. The *X*-*axis* shows the index of 90 pathways, and the *Y*-*axis* shows the number of edges and nodes in each pathway

The 90 soybean-related metabolic pathways were downloaded from the KEGG database with the assumption that they represent physiology-relevant pathways in order to validate the pathway prediction results. The statistics of nodes and the edge relationships (e.g., ECrel, PPrel, GErel) for each target pathway are shown in Fig. 3, along with the number of true soybean genes mapped to each target pathway. Nine pathways with no detected edges were removed giving a total of 81 pathway sets for validation and evaluation of the performance of Bayesian network construction.

**Fig. 4:**
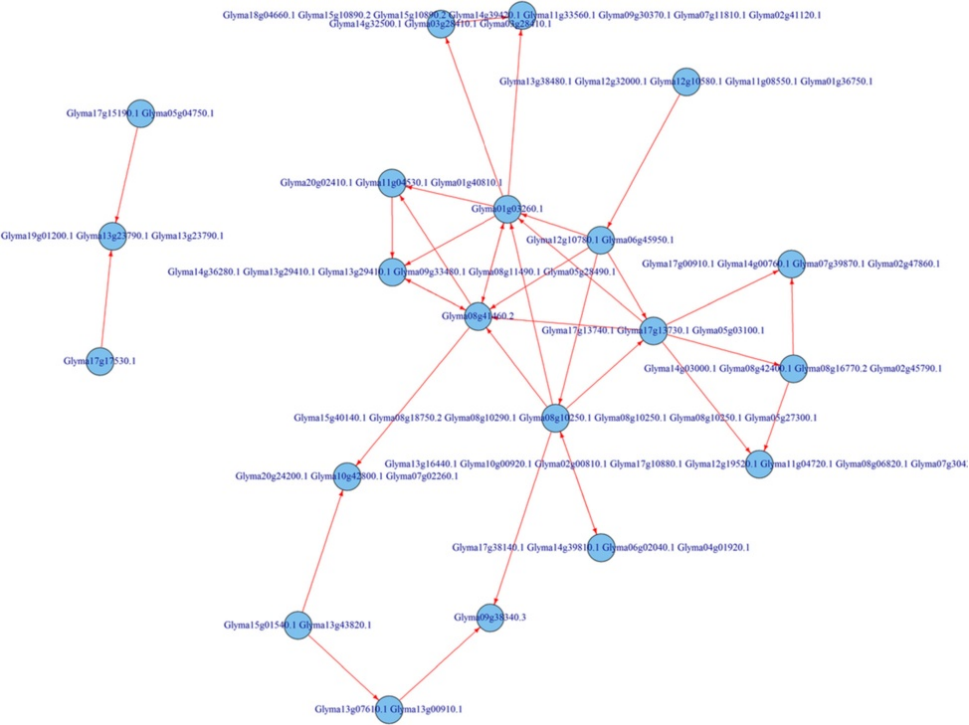
Initial mapping for the glyoxylate and dicarboxylate metabolism pathway (KO00630). Each *node* in the figure denotes the genes mapped to the orthology group in the glyoxylate and dicarboxylate metabolism pathway from KEGG. The *edges* (*red color*) denote the true edges that exist in the target pathway in KEGG

The KEGG Automatic Annotation Server (KAAS) mapped all 26,873 soybean genes based on the assigned KO number. During KO-gene assignment in the initial mapping process, soybean-related gene information and pathways in the KEGG database were excluded in order to validate our results back to these reference pathways. Among the 26,873 genes, 9272 genes were successfully assigned to KO identifiers in KEGG. The network structure for each pathway can be downloaded and viewed using the KEGG Markup Language (KGML), which is XML format based. KGML represents the pathway as a graph object comprised of the entry nodes labeled as K numbers and edges with the relation and reaction elements. By way of example, the glyoxylate and dicarboxylate metabolism pathway (KO00630) is shown in Fig. 4.

### Metabolic pathway prediction

**Fig. 5:**
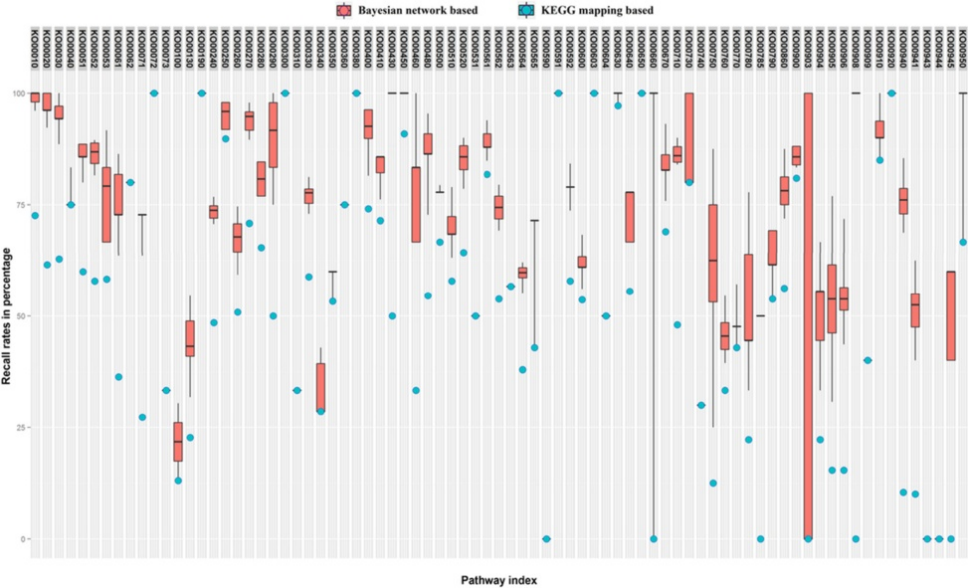
Recall rate for two methods. Recall rate (i.e., the rate of the edges in target pathways that were correctly mapped) using two methods: 1. Traditional mapping method using KASS. 2. Probabilistic graphical model and knowledge-based method. The recall rate is calculated by the division of the number of edges mapped in target pathway and the total number of edges in target pathway. The experiment was repeated 50 times on each pathway and the average recall rate was calculated

**Fig. 6:**
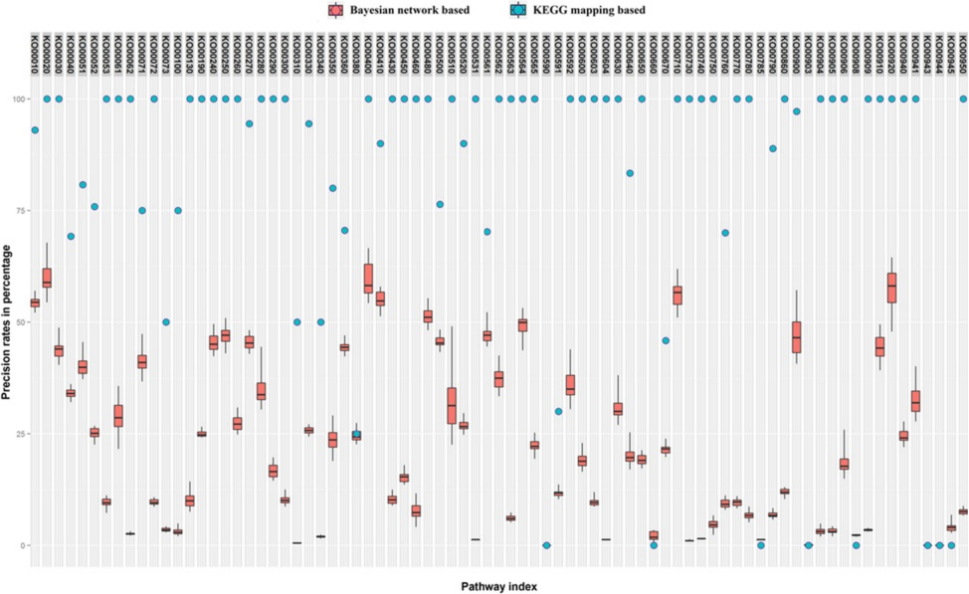
Precision rate for two methods. Precision rate (i.e., the rate of edges in predicted pathways that were mapped to target pathways) using two methods: 1. Traditional mapping method using KASS. 2. Probabilistic graphical model and knowledge-based method. The precision rate is calculated by the division of the number of edges in predict pathways that exists in target pathways and the total number of edges in predicted pathway. The experiment was repeated 50 times on each pathway and the average recall rate was calculated

In order to evaluate the prediction accuracy for the probabilistic network method using the soybean data, we applied the method on the 81 reference pathways to predict new networks. The 81 new networks were compared to the target pathways downloaded from the KEGG database in terms of the recall rate (i.e., the rate of the edges in target pathways that were correctly mapped) and precision rate (i.e., the rate of edges in predicted pathways that were mapped to target pathways) [9]. The experiment was repeated 50 times on each pathway and the average recall rate and precision rate are shown in Figs. 5 and 6, respectively. As demonstrated in Fig. 5, the probabilistic and knowledge-based method predicted more true relations than the initial mapping methods in terms of recall rate. This is due to the initial exclusion of the soybean-related gene information from the KEGG database when performing the gene-to-KO assignments through KAAS, which allowed consideration of gene information across all the other species. Hence, some true KO numbers were not assigned to genes correctly, which led to incomplete assignments during the initial mapping. After applying the Bayesian network based on the data-driven and knowledge-driven methods, the new relationships could be predicted successfully. Figure 6 showed that our method can also predict new gene relationships that do not exist in the reference pathway, in addition to completely incorporating the initial mapping results (i.e., no information is loss from the initial KAAS-generated pathways).

**Fig. 7:**
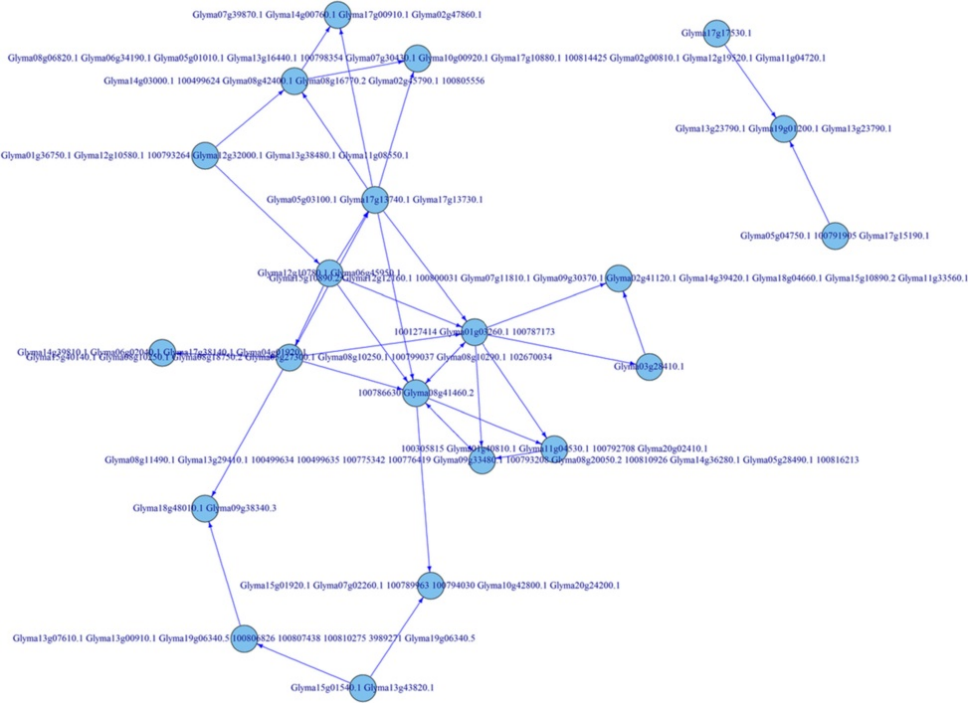
Target pathway of the glyoxylate and dicarboxylate metabolism pathway (KO00630) in KEGG. Each *node* in the figure denotes the soybean genes annotated in the glyoxylate and dicarboxylate metabolism pathway from KEGG. The *edges* between genes denote all the true edges that exist in the target pathway in KEGG

**Fig. 8:**
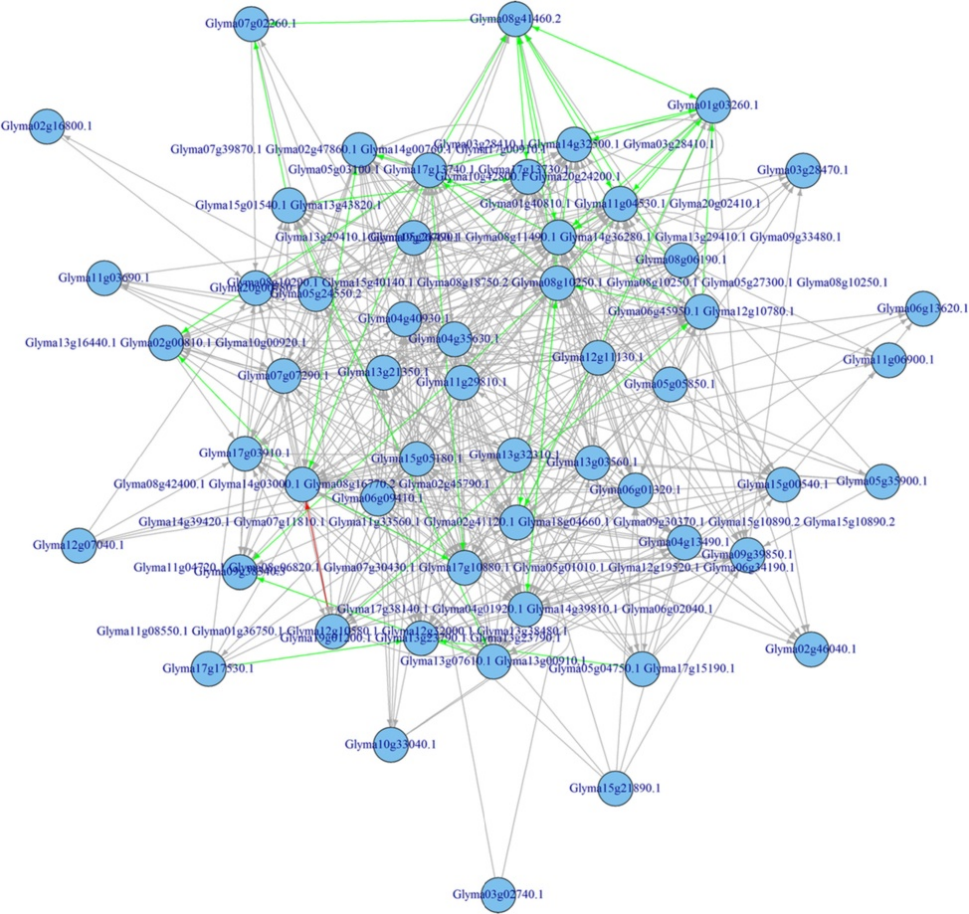
Predicted graphical network model. Predicted network pathway for the glyoxylate and dicarboxylate metabolism pathway (KO00630) was constructed based on the probabilistic network modeling method. The *green edges* denote the relations that exist in the initial mapping pathway. The *red edge* denotes the predicted true edge that exists in the target pathway. This figure showed that the true relation was predicted successfully from the initial mapping

**Fig. 9:**
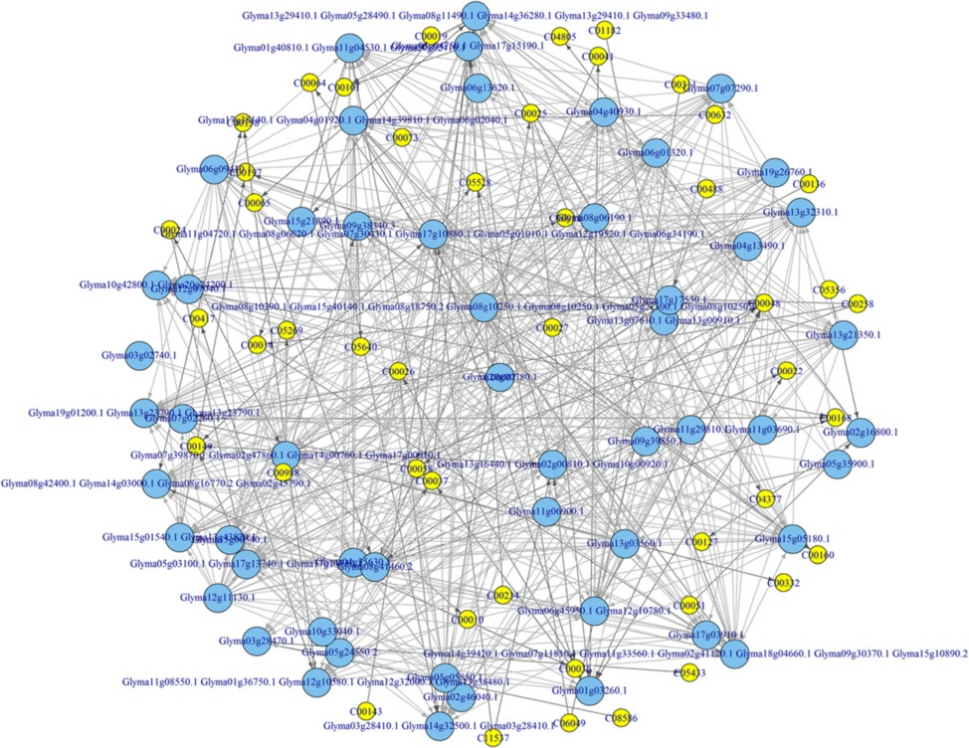
Final predicted metabolic pathway for the glyoxylate and dicarboxylate metabolism pathway (KO00630). The metabolic pathway was reconstructed for the glyoxylate and dicarboxylate metabolism pathway (KO00630) based on the probabilistic network modeling method with inclusion of the associated chemical compounds. The *yellow nodes* denote the chemical compounds extracted from the KEGG knowledgebase

Again, using the glyoxylate and dicarboxylate metabolism pathway (KO00630) as an example, the whole process for the metabolic pathway construction can be viewed through Figs. 4, 7, 8, and 9. Figure 4 represents the initial mapping for the pathway and, when compared to the true target pathway shown in Fig. 7, the initial mapping method does not predict all the edges in the target pathway. Applying the probabilistic modeling method by adding more genes into the pathway creates a more complete network, and all the true relationships in the target pathway are predicted successfully, which is shown in Fig. 8. The green-colored edge denotes the edge existing in the target pathway, and the edge with red color represents the edge not included in the initial mapping network while involved in the target pathway. Figure 9 shows the final metabolic pathways by attaching related chemical reactions. The new method has the capability to predict new relationships and reactions between genes and improve the prediction accuracy compared to the traditional reference pathway mapping approach.

### Function enrichment analysis

**Fig. 10:**
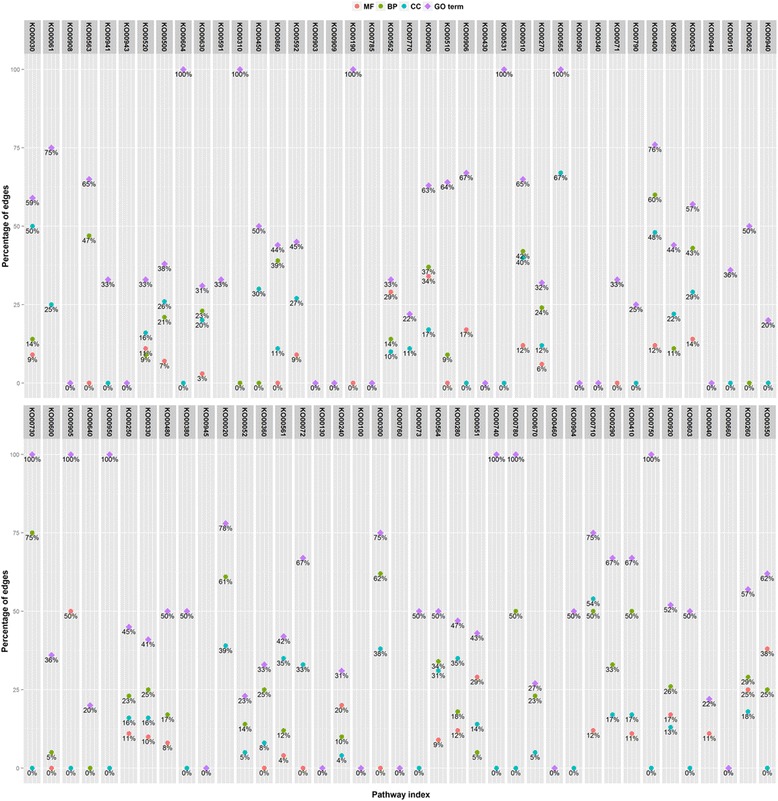
The distribution of edges mapped to each category of Gene Ontology and GO terms in the initial mapping pathway. The percentage of edges in the initial mapping pathway which were related to each GO category (e.g., molecular function, biological process, and cellular component) was calculated. The proportion of gene pairs that shared identical GO terms in each pathway was also provided

**Fig. 11:**
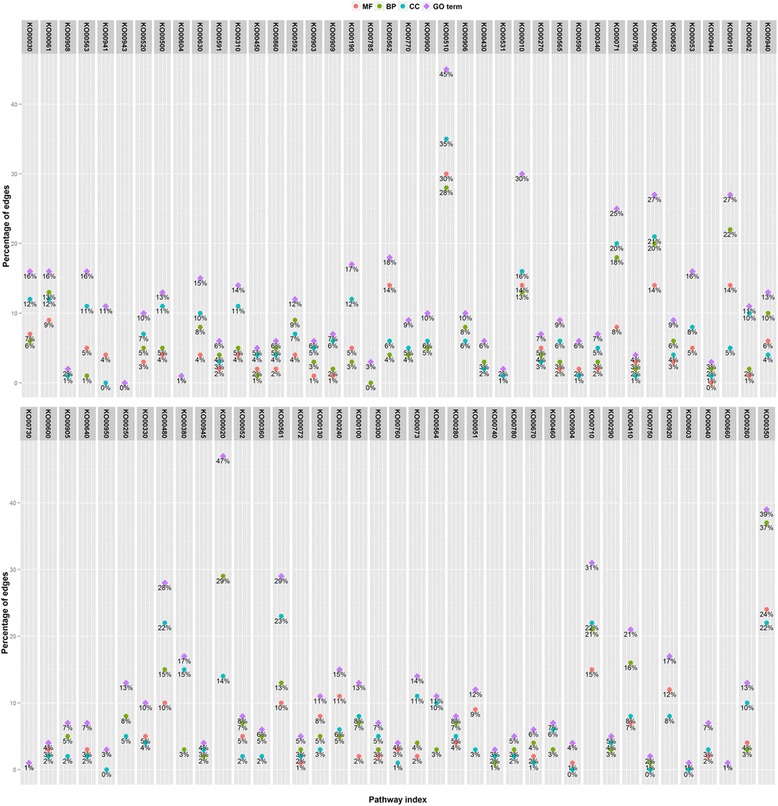
The distribution of predicted edges mapped to each category of Gene Ontology and GO terms in the Bayesian network pathway. The percentage of predicted edges in the Bayesian network pathway which were related to each GO category (e.g., molecular function, biological process, and cellular component) was calculated. The proportion of predicted gene relations that shared identical GO terms in each pathway was also provided

MULTICOM-PDCN [16, 17] was used to predict functions of 26,873 genes that were used in the whole pathway construction. For the 26,873 genes, 5938 GO terms were identified, with 54.02, 33.05, and 12.86 % GO terms belong to biological process, molecular function, and cellular component, respectively. Gene function enrichment analysis was conducted on each pathway to identify significantly enriched functions based on the Fisher exact test using a *P* value less than 0.05. The predicted edges in each pathway were validated in two ways: (1) the respective percentage of predicted gene pairs belong to the three categories (i.e., biological process, molecular function, and cellular component) and (2) the percentage of predicted gene pairs mapped to the same GO terms. Figure 10 reports the distribution of edges related to the three GO categories and GO terms in the initial pathway from the KEGG database. Figure 11 represents the distribution of predicted edges in which the initial mapped edges were excluded in the final reconstructed metabolic pathway associated with the three GO term categories. Recent studies found that genes in the same metabolic pathway are more likely to be co-expressed [23, 24]. By assuming genes with a similar expression structure may have similar function properties, we tested whether gene pairs in the same metabolic pathway share identical GO terms or belong to the same GO categories, which we used to further validate our network. As shown in Fig. 10, 35.69 % of 15 edges on average across all the 81 initial mapping pathways from KEGG shared the same GO annotations, in which 17, 29.16, and 16.79 % of gene-set pairs show the significant enrichment for molecular function, biological process, and cellular components, respectively. Therefore, as expected, gene pairs connected in metabolic pathways often shared similar functional properties, even though not all of the genes in the same pathways have identical GO annotations [23, 24]. In Figure 11, among all the predicted edges, an average of 9.17 % of 384 gene-set pairs have identical GO annotation, with 4.13, 5.24, and 5.27 % of gene-set pairs associated to molecular function, biological process, and cellular components, respectively. The top 10 enriched functions in the predicted Bayesian network for the glyoxylate and dicarboxylate metabolism pathway are listed in the Table 1. Several functions, such as glyoxylate cycle, l-malate dehydrogenase activity, and malate metabolic process are over-represented in this glyoxylate pathway. Two gluconeogenesis and the peroxisomal glyoxylate cycle-related gene Glyma14g03000.1 (citrate synthase) and Glyma11g04720.1 (NAD-dependent malate dehydrogenase) were predicted to be linked in the reconstructed metabolic pathway, while this edge was not mapped successfully in the initial mapping pathway but clearly represents a true relationship in the target pathway. The gene Glyma17g13730.1 (malate synthase) and Glyma06g45950.1/Glyma12g10780.1 (isocitrate lyase) were also paired with a forward direction in the final predicted network.

**Table 1:** The top 10 enriched functions of 106 genes identified in the predicted network for the glyoxylate and dicarboxylate metabolism pathway (KO00630)

GO term	Category	Functions	*P* value
GO:0006097	Biological process	Glyoxylate cycle	2.20E-16
GO:0006099	Biological process	Tricarboxylic acid cycle	2.20E-16
GO:0009514	Cellular component	Glyoxysome	2.20E-16
GO:0030060	Molecular function	l-Malate dehydrogenase activity	2.20E-16
GO:0006542	Biological process	Glutamine biosynthetic process	5.01E-14
GO:0004356	Molecular function	Glutamate-ammonia ligase activity	2.46E-13
GO:0006108	Biological process	Malate metabolic process	1.93E-12
GO:0004460	Molecular function	l-Lactate dehydrogenase (cytochrome) activity	1.35E-11
GO:0006089	Biological process	Lactate metabolic process	1.35E-11
GO:0055114	Biological process	Oxidation reduction	1.52E-10

## Conclusions

In this study, we applied probabilistic graphical and knowledge-based methods to reconstruct soybean metabolic pathways based on a comprehensive transcriptome database. Based on the results, the method performed better than the traditional sequence-homology mapping method by predicting more real relationships in the pathways. Functional enrichment analysis on the predicted pathways also revealed that functional related gene pairs were predicted successfully to enlarge the initial mapping network from KEGG. The good performance of the data and knowledge-based probabilistic method provided fundamental, new biological information for soybean research and demonstrates that this method can be generally applicable for other genomes where similar starting data are available.
